# Crosstalk between autophagy and insulin resistance: evidence from different tissues

**DOI:** 10.1186/s40001-023-01424-9

**Published:** 2023-10-25

**Authors:** Asie Sadeghi, Maryam Niknam, Mohammad Amin Momeni-Moghaddam, Maryam Shabani, Hamid Aria, Alireza Bastin, Maryam Teimouri, Reza Meshkani, Hamed Akbari

**Affiliations:** 1 Student Research Committee, Kerman University of Medical Sciences, Kerman, Iran; 2 Department of Clinical Biochemistry, Faculty of Medicine, Kerman University of Medical Sciences, Kerman, Iran; 3 Department of Biochemistry, School of Medicine, Shiraz University of Medical Sciences, Shiraz, Iran; 4 Department of Nutrition and Biochemistry, Gonabad University of Medical Sciences, Gonabad, Iran; 5 Department of Clinical Biochemistry, Faculty of Medicine, Tehran University of Medical Sciences, Tehran, Iran; 6 Noncommunicable Diseases Research Center, Fasa University of Medical Sciences, Fasa, Iran; 7 Department of Immunology, School of Medicine, Isfahan University of Medical Sciences, Isfahan, Iran; 8 Clinical Research Development Center “The Persian Gulf Martyrs” Hospital, Bushehr University of Medical Sciences, Bushehr, Iran; 9 Department of Biochemistry, School of Allied Medical Sciences, Shahroud University of Medical Sciences, Shahroud, Iran

**Keywords:** Adipose tissue, Autophagy, Insulin resistance, Diabetes complications, Type 2 diabetes

## Abstract

Insulin is a critical hormone that promotes energy storage in various tissues, as well as anabolic functions. Insulin resistance significantly reduces these responses, resulting in pathological conditions, such as obesity and type 2 diabetes mellitus (T2DM). The management of insulin resistance requires better knowledge of its pathophysiological mechanisms to prevent secondary complications, such as cardiovascular diseases (CVDs). Recent evidence regarding the etiological mechanisms behind insulin resistance emphasizes the role of energy imbalance and neurohormonal dysregulation, both of which are closely regulated by autophagy. Autophagy is a conserved process that maintains homeostasis in cells. Accordingly, autophagy abnormalities have been linked to a variety of metabolic disorders, including insulin resistance, T2DM, obesity, and CVDs. Thus, there may be a link between autophagy and insulin resistance. Therefore, the interaction between autophagy and insulin function will be examined in this review, particularly in insulin-responsive tissues, such as adipose tissue, liver, and skeletal muscle.

## Introduction

### Insulin resistance

Type 2 diabetes mellitus (T2DM) is characterized by pathophysiological abnormalities, including impaired insulin secretion from pancreatic islet β-cells, peripheral insulin resistance, and excessive glucose production by the liver. When tissues such as muscle, fat, and liver fail to react properly to insulin, glucose is not readily eliminated from the blood; as a result, the tissues begin to exhibit insulin resistance, which causes the pancreas to produce more insulin in order for the cells to receive glucose [1, 2]. Additionally, pathophysiological conditions such as hyperglycemia, hypertension, visceral adiposity, dyslipidemia, hyperuricemia, elevated inflammatory markers, endothelial dysfunction, and a prothrombic state are metabolically caused by insulin resistance. If insulin resistance continues to increase, it will lead to T2DM, nonalcoholic fatty liver disease (NAFLD), and metabolic syndrome [2]. Decreased insulin binding to its receptor, receptor phosphorylation, tyrosine kinase activity, phosphorylation of insulin receptor substrates (IRSs), and GLUT4 downregulation are observed in insulin-resistant tissues [3]. Other modifications specific to certain tissues will also occur, including changes in the adipocytes of obese individuals with T2DM, decreased insulin receptor substrate (IRS-1) expression, reduced activity of IRS-1-associated phosphoinositide 3-kinase (PI3K), and IRS-2 becoming the main docking protein for PI3K [4]. Nonetheless, it should be noted that even though protein levels of IRS-1 and IRS-2 are normal, the PI3K function linked with both IRSs is impaired in the skeletal muscle of obese T2DM patients [5].

Insulin resistance in obese individuals is caused by a number of mechanisms. As suggested by several studies, proinflammatory cytokines and endoplasmic stress contribute to insulin resistance by activating serine kinases c-Jun N-terminal kinase (JNK) and I kappa B kinase (IKK-b), which are responsible for increasing the phosphorylation of IRS1 at serine sites (serine 302 pS302 and serine 307 pS307). This, in turn, leads to negative regulation of normal insulin signaling [6]. High levels of adipocyte lipid deposition in obese individuals lead to the production of proinflammatory cytokines including interleukin (IL)-6, IL-1b, tumor necrosis factor-alpha (TNF-α), and resistin, resulting in further activation of JNK and nuclear factor kappa B (NF-κB) pathways [7, 8]. In obesity, an increase in fatty acids and their intracellular metabolites (e.g., diacylglycerols) stimulates protein kinase C (PKC), resulting in the activation of serine/threonine kinases and impaired tyrosine phosphorylation of IRS [9, 10].

As major metabolic organelles, mitochondria play a significant role in the insulin signaling of dependent tissues. Insulin is not only required for mitochondria to function properly, but also serves as the basis for the integrity of the mitochondrial electron transport chain (mETC) as it damps down forkhead box O (FOXO) 11/heme oxygenase (HMOX) 12 and maintains a consistent NAD/NADH ratio in mitochondria [11, 12]. Oxidative stress is shown to cause insulin resistance by impairing insulin signal transduction and dysregulating adipokines [13]. In addition, it contributes to the activation of several serine-threonine kinase pathways namely inhibitor of nuclear factor kappa-B kinase subunit beta (IKKβ)/NF‐κB and JNK. These pathways, in turn, lead to the phosphorylation of IRS proteins and consequent IRS degradation [14]. It has been demonstrated that high levels of free radicals suppress the cell membrane GLUT‐4 localization by impairing insulin signaling [15].

Other associations between oxidative stress and insulin resistance include reduced insulin‐caused relocation of IRS‐1 and phosphatidylinositol (PIP) kinase between microsomes and cytoplasm, decreased protein kinase B (PKB) phosphorylation, serine phosphorylation at the serine 307 of IRS‐1, and lowered GLUT‐4 expression [14, 15]. Moreover, it has been found that β-cell endoplasmic reticulum (ER) stress leads to the disruption of insulin synthesis and sensitivity [16, 17].

## Autophagy

Autophagy or “self-eating” is a conserved process essential for sustaining cellular homeostasis. This process allows cells to degrade and remove aggregated or misfolded proteins, damaged organelles, and pathogens [18]. During the autophagy pathway, cellular components are transported to lysosomes to be degraded and recycled. There are three main types of autophagy based on how substrates are delivered to the lysosome: chaperon-mediated autophagy, micro-autophagy, and macroautophagy [19]. Since macroautophagy is the preliminary model of autophagy, similar to previous research, we reserve the term autophagy to refer to this type. The components of autophagy machinery and its basic mechanisms were first explored in *Saccharomyces cerevisiae*, and subsequently, they were studied in other organisms, particularly in mammalians [20]. The process of autophagy generally involves the following steps: induction, formation of the initial nucleus, development of autophagosomal vesicles, combination with lysosomes, and finally digestion. Most proteins implicated in this process are known as autophagy-related (ATG) proteins [21].

**Fig. 1:**
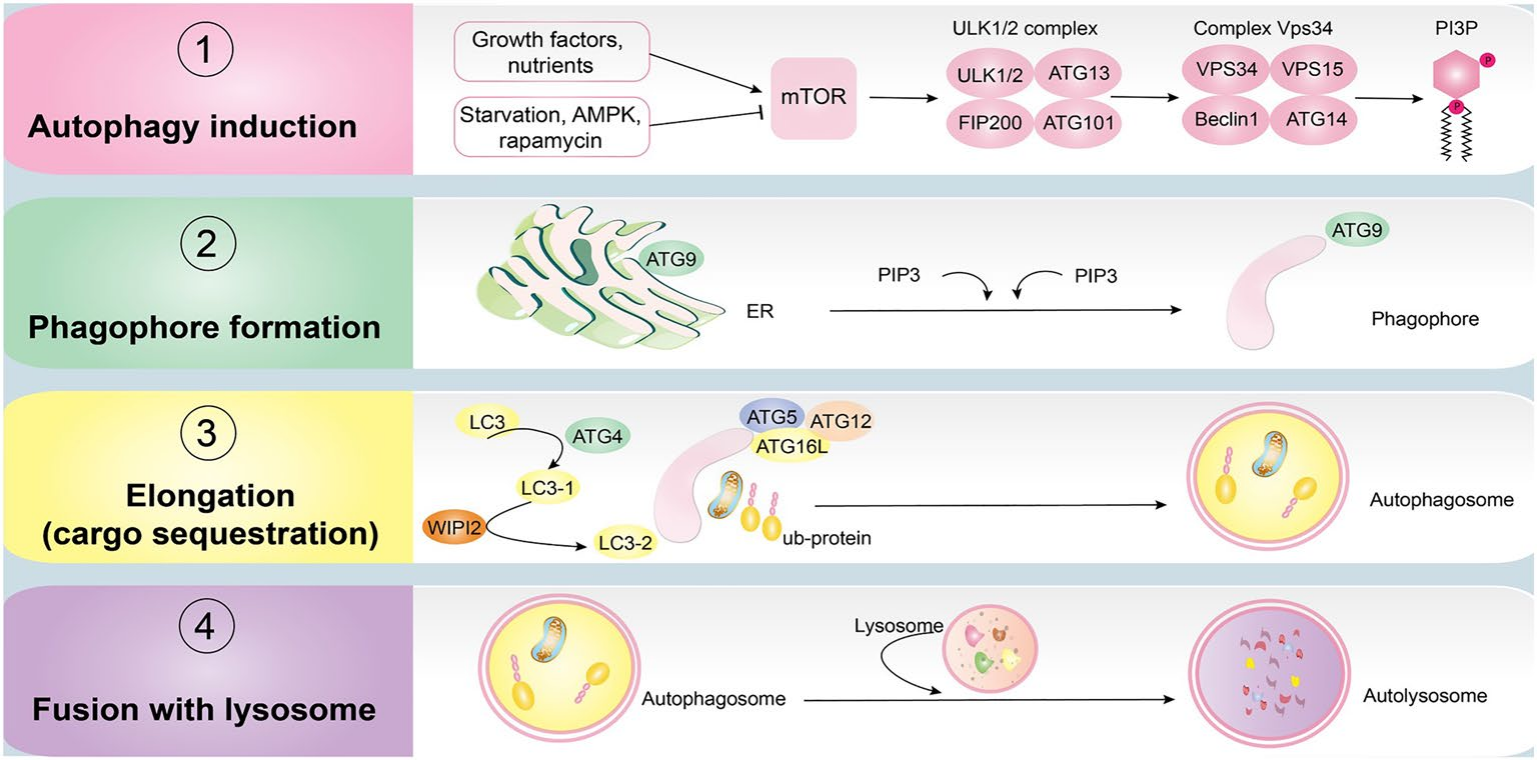
The mechanism of the autophagy pathway in mammalian cells. The autophagy process includes four main steps: induction, phagophore formation, phagophore elongation, and fusion with the lysosome. In the first step, different signals, such as starvation, rapamycin, and similar factors, inhibit mTOR and activate the ULK complex, thereby inducing autophagy. Active ULK stimulates the Vps34 complex to produce PI3P, which is needed for phagophore formation. In the following step, the phagophore expands, engulfs cytoplasmic constituents, and finally fuses with the lysosome to degrade their contents

In mammalians, the Unc-51-like kinase 1/2 (ULK1/2) complex consisting of ULK1/2, ATG13, FIP200, and ATG 101 is involved in the induction of autophagy [22]. Under conditions of high nutrients or in the presence of growth factors, the mammalian target of rapamycin (mTOR) complex 1 (mTORC1) phosphorylates and inactivates ULK1/2 and ATG13, resulting in the inhibition of autophagy. During starvation (especially amino acid depletion) or in the presence of rapamycin (a specific inhibitor of mTOR), however, mTORC1 disassociates from ULK1, thereby inducing autophagy [22]. Another energy sensor is AMP-activated protein kinase (AMPK), which activates autophagy when ATP levels drop, either directly by phosphorylating ULK1 or indirectly by inactivating mTORC1 [23]. Active ULK then stimulates class III PI3K or the vacuolar protein sorting 34 (Vps34) complex and recruits it to the phagophore assembly site (PAS) [24]. The Vps34 complex is composed of Vps34, beclin-1, Vps15, ATG14, and activating molecule in beclin-1-regulated autophagy (AMBRA1) [24]. This complex synthesizes phosphatidylinositol triphosphate (PI3P), which is required for phagophore localization. Phagophore is a special membrane that starts the sequestration of cytoplasmic constituents. Elongation of phagophore to a double membrane vesicle (called autophagosome) completes the sequestration [25]. Two conjugation systems promote autophagosome formation: ATG5-ATG12-ATG16L and microtubule-associated protein 1 light chain 3 (LC3). LC3-I is a cleaved form of LC3 which is converted to LC3-II by lipidation as autophagy progresses [19]. A rise in LC3-II levels, according to the increased number of autophagosomes, suggests autophagy induction. LC3 serves as a receptor for adaptor protein p62/sequestosome 1 (SQSTM1), which can interact with ubiquitinated substrates, and make them a target for lysosomes [26]. The autophagy pathway is completed by the fusion of lysosomes and autophagosomes, leading to the formation of autophagolysosomes whose contents are degraded and recycled. P62 itself is degraded in autophagolysosomes and since its level is used as a tool to assess the autophagic flux, an increase in p62 levels denotes autophagy flux inhibition and vice versa [26] (Fig. 1).

**Fig. 2:**
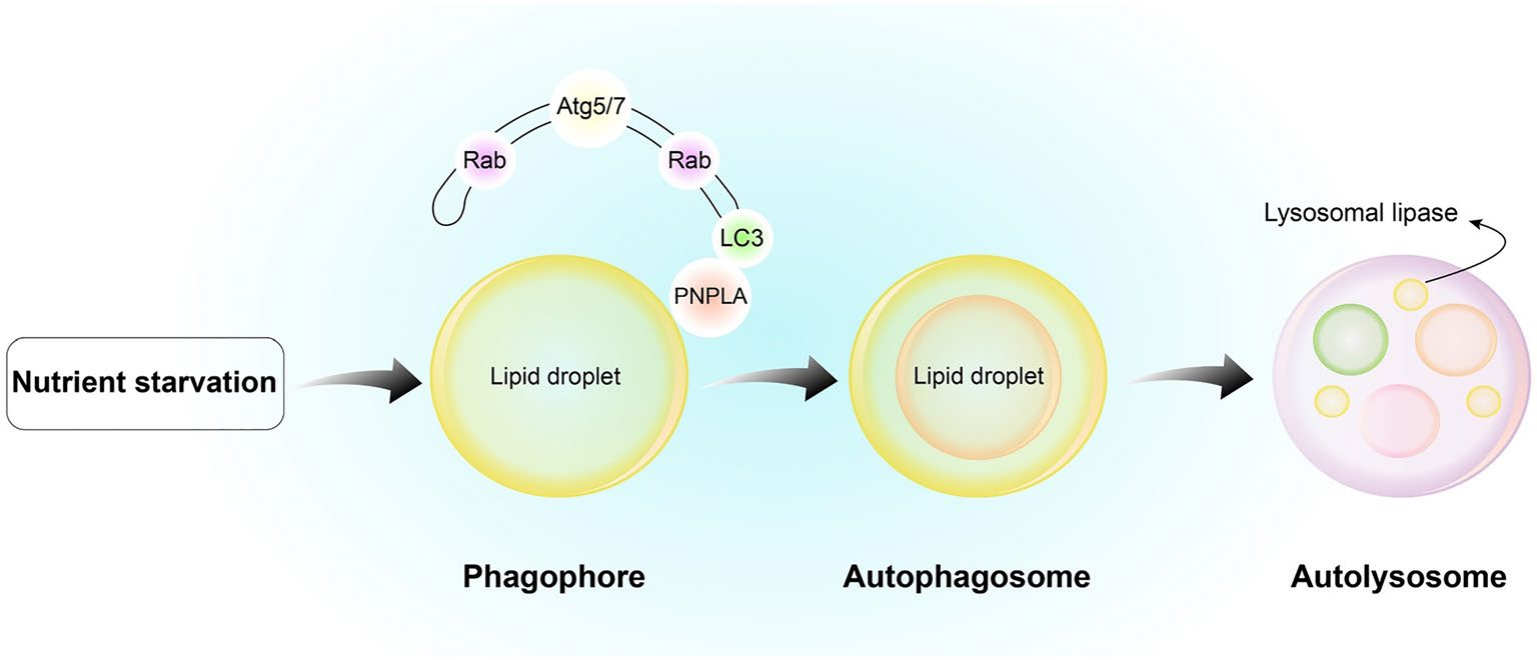
Molecular mechanism of lipophagy. Under nutrient deprivation, lipophagy forms phagophores. Patatin-like phospholipase domain-containing enzyme (PNPLA) interacts with LDs and performs critical functions in the breakdown of LDs. Autophagosomes engulf LDs and fuse with a lysosome to form an autolysosome. Then, lysosomal lipases hydrolyze the neutral lipids of LDs

Autophagy is generally considered a defense mechanism against various stresses, such as nutrient deficiencies or caloric restriction (the most well-known), oxidative injury, and depletion of growth factors, and it promotes cell survival. In unfavorable conditions, it provides nutrients for cells and removes toxic molecules, damaged organelles, and infectious agents [19]. In addition to the maintenance of homeostasis, autophagy regulates the maturation, development, and differentiation of various cells, each with a physiological role (e.g., adipocytes in fat metabolism) [27]. Accordingly, the impairment of this process is associated with pathological conditions including cancer, inflammation, and autoimmunity, as well as metabolic, cardiovascular, and neurodegenerative disorders [28, 29]. Furthermore, autophagy gradually subsides with aging, which is attributed to age-associated diseases such as T2DM [30]. One of the main characteristics of T2DM is insulin resistance. In this review, we discuss the role of autophagy in the target tissues of insulin with a focus on its contribution to the pathophysiology of insulin resistance (Fig. 2).

## Adipose tissue

### Altered adipose tissue autophagy in obesity

Obesity, characterized by a remarkable expansion of adipose tissue mass, is the leading risk factor for insulin resistance. Based on previous studies, human adipose tissue and adipocytes both contain autophagosomes and express autophagy genes [31]. Ost et al. originally revealed that the autophagosome content and the autophagic flux were enhanced in adipocytes isolated from obese individuals [32]. This observation was confirmed by studies that reported an enhanced expression of autophagic machinery genes in the adipose tissue of obese patients, such as autophagy-related genes (*ATG5, ATG7*, and *ATG12*), beclin-1, LC3A and B, LC3-II, and p62. This increased expression was attributed to the enhanced improved function of both the E2 factor (E2F) and FOXO transcription factors [33, 34]. Interestingly, it has been reported that upregulation of autophagy in obese individuals is more pronounced in omental fat tissue than in subcutaneous fat tissue. Consistently, higher expression of autophagy-related genes was shown in the visceral fat depot, which is linked to metabolic and cardiovascular risks [35, 36]. The rates of fat accumulation and hypertrophy of fat cells are significantly associated with the expression of autophagy genes, and autophagy alteration is accompanied by obesity-associated insulin resistance prior to metabolic and cardiovascular dysfunction [31].

In addition, ER stress, inflammation, and hypoxia, which are the processes that are active in adipose tissue during obesity, promote autophagy via the suppression of mTORC1 [37]. On the whole, autophagy could serve as a protective mechanism against increased inflammation associated with obesity or as a compensatory response to the excessive accumulation of nutrients and damaged organelles in hypertrophic adipocytes [38].

### Autophagy, insulin signaling, and insulin resistance in adipose tissue

**Fig. 3:**
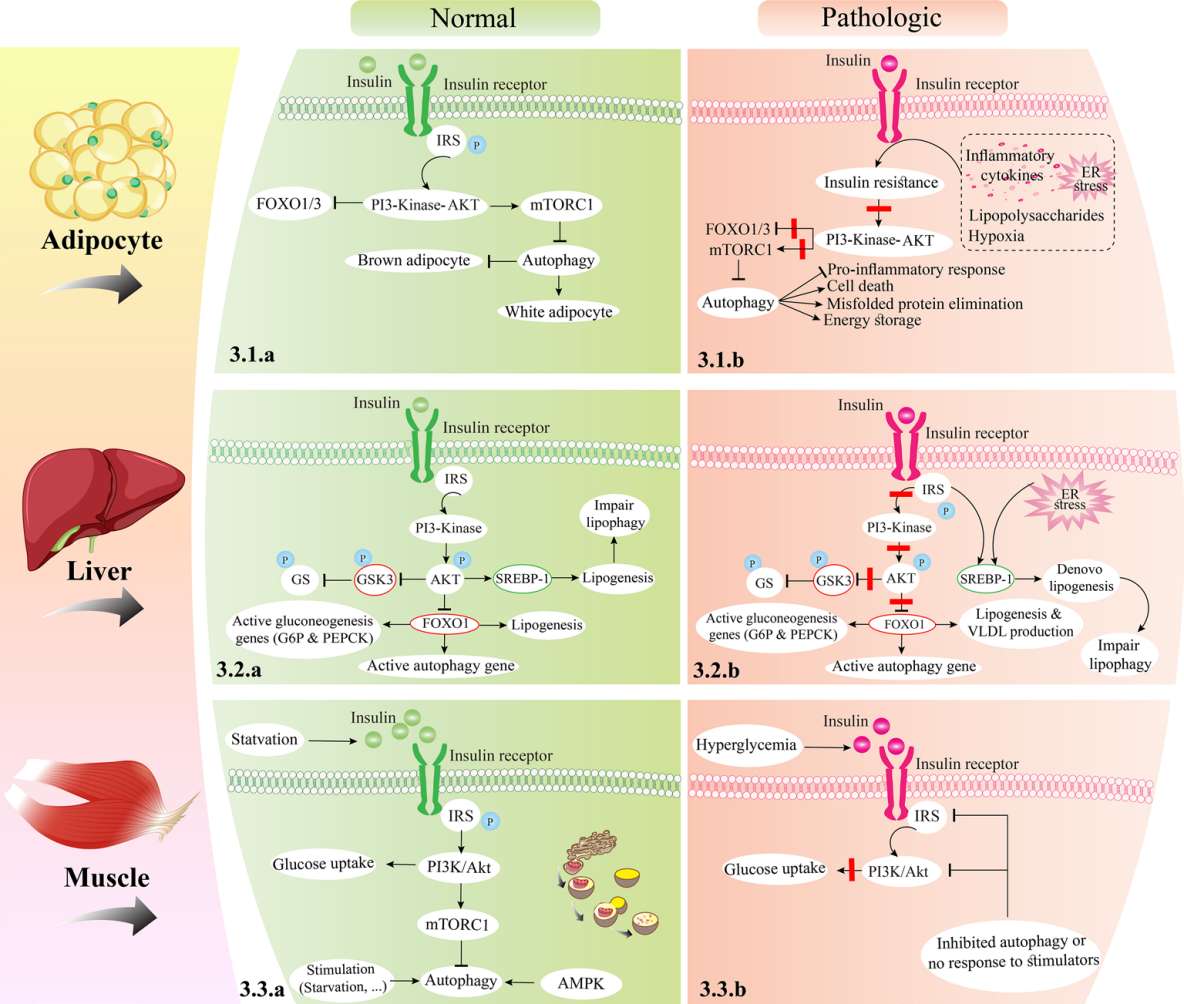
The insulin signaling under physiological and pathological conditions in various organs. **3.1**: autophagy regulation in lean and obese adipose tissue and its effects on adipocytes. In the lean state (**3.1.a**), the stimulation of mTORC1 by insulin results in to autophagy inhibition. The inhibition of autophagy induces the ‘browning’ phenotype of the adipocytes. In obesity (**3.1.b**), ER stress, hypoxia and inflammation stimulate insulin resistance, resulting in mTORC1 inhibition and subsequently to induction of autophagy. Autophagy improve adipocyte function through eliminating damaged organelles and misfolded proteins and prohibiting the proinflammatory responses. Furthermore, excessive stimulation of autophagy may increase energy storage of adipocyte and promote cell death. **3.2**: in physiological state (**3.2.a**), following the binding of insulin to its receptor, Akt is activated and leads to the inhibition of glycogen synthase kinase 3 and forkhead box O (FOXO) 1 in liver. Inhibition of glycogen synthase kinase leads to increased glycogen synthase activity, therefore, increased glycogen synthesis. Following the inhibition of FOXO1, the expression of glucose 6 phosphatase and phosphoenol pyruvate carboxykinase genes decreases, as a result, decreases the hepatic glucose production. Also, insulin decreases autophagy by activating Akt and inhibiting FOXO1. Akt increases lipid synthesis in hepatocytes by activating SREBP1c. The increase in lipid content by SREBP1c causes disruption in lipophagy and hepatic steatosis. In insulin resistance state (**3.2.b**), glycogen synthase kinase is activated and inhibits glycogen synthase. FOXO1 activity is increased, subsequently, gluconeogenesis pathway, lipid synthesis and very-low-density lipoprotein (VLDL) as well as autophagy are activated. SREBP1c increases its activity through ER stress or through IRS-1. **3.3**: in the physiological state during starvation (**3.3.a**), the insulin hormone binding to its receptors causes the signaling adapter IRS-1 to be recruited and activate AMPK. Active AMPK causes mTORC1 reduction and an increase in autophagy. In a pathological state and during hyperglycemia (**3.3.b**), the binding of insulin to its receptor causes IRS-1 phosphorylation and recruits PI3K in muscles. Then, the PI3K converts PIP2 to PIP3, as a result, PIP3 induces Akt phosphorylation and increases glucose uptake. Also, Akt phosphorylation increases mTORC1 and decreases autophagy. mTORC1; mammalian target of rapamycin complex 1, ER; endoplasmic reticulum, FOXO1; forkhead box O1, SREBP1c; sterol regulatory element-binding transcription factor 1, VLDL; very-low-density lipoprotein, IRS-1; insulin receptor substrate 1, AMPK; AMP-activated protein kinase, IR; insulin receptor, PI3K; phosphatidylinositol-3-kinase, PIP2; phosphatidylinositol 4,5-bisphosphate, PIP3; phosphatidylinositol-3, 4, 5-triphosphate

Insulin, an anabolic hormone, is a potent inhibitor of autophagy [39]. It has been demonstrated that insulin can prevent autophagy by mTORC1 activation, resulting in the suppression of FOXO and ULK1 factors [40]. The PI3K–Akt pathway is a crucial constituent of insulin signaling, which contributes to the inhibition of autophagy by insulin. Akt inhibits FOXO1/3 and induces mTORC1 activity, thereby revealing a major association between insulin signaling and autophagy [34, 41]. However, targeted *ATG7* deletion causes detrimental differentiation of WAT, and its browning, resulting in improved insulin sensitivity, and glucose use, as well as enhanced β-oxidation of fatty acids [42, 43]. These differentiations and metabolic changes by autophagy inhibition have a critical role in insulin resistance [34]. However, in a study by Cai et al. on mouse models, specific knockout of *ATG3* and *ATG16L1* in adipocytes caused insulin resistance. Inhibiting autophagy in the adipocytes, in particular, interfered with insulin signaling to Akt in the WAT, liver, and skeletal muscle [44]. As cells that highly infiltrate into WAT during obesity, macrophages can affect insulin sensitivity and glucose homeostasis [45]. Previous studies have demonstrated that the knockout of *ATG5* or *ATG7* in macrophages interferes with insulin sensitivity and glucose tolerance [46, 47]. Figure 3 illustrates how autophagy is regulated in both lean and obese adipose tissue, as well as how it affects adipocytes (3.1.a and 3.1.b). Based on these findings, it could be concluded that congenital autophagy inhibition impairs adipogenesis and leads to insulin sensitivity, whereas selective inhibition of this pathway in mature adipocytes results in insulin resistance [34].

### Autophagy and adipose tissue development and its contribution in insulin resistance

Adipogenesis is a two-step process in which fibroblast-like, multipotent adipose mesenchymal stem cells (ASCs) are converted to mature adipocytes [33]. As the most frequent cells in adipose tissue, adipocytes are involved in storing energy as fat. During obesity, an increase in the size and number of these cells leads to the expansion of adipose tissue, which is closely connected to insulin resistance [48].

It has been demonstrated that autophagy is involved in the development and differentiation of adipose tissue. Meanwhile, the exact regulatory mechanisms of autophagy in adipogenesis are unknown. As a possible mechanism, autophagosomes facilitate the reorganization of cytoplasmic components via the mobilization of membranes within the cell, thereby contributing to adipogenesis [49]. Furthermore, it has been shown that the stability of peroxisome proliferator-activated receptor (PPAR) γ, which is a major regulator of adipogenesis and adipocyte differentiation, is increased by autophagy [50].

It has been revealed that genetic interference with the process affect pathways linked to adipose differentiation. As an example, *ATG5* defect in pre-adipocytes, prevents complete adipocyte differentiation and results in the characteristics of adipose tissue brown fat [42, 51, 52]. In this regard, it has been reported that in *ATG5*^−/−^ mice, mouse embryonic fibroblasts (MEFs) showed reduced adipogenesis. These cells exhibited signs of adipogenesis initiation and triacylglycerol accumulation, but were not able to complete the maturation of adipocytes [51]. It has also been reported that in mice with the adipose specific *Atg7* knockout, the amount of BAT is increased. In fact, in lack of autophagy, progenitor cells fail to complete differentiation and so follow default pathway and create BAT. BAT indicators such as peroxisome proliferator-activated receptor-gamma coactivator-1 alpha (PGC-1α), UCP-1, and mitochondrial enzymes are increased, which is associated with weight loss and increases β-oxidation. BAT development in these animals enhanced insulin sensitivity in body, demonstrating adipose tissue autophagy has a large effect on insulin sensitivity [42]. Furthermore, based on in vivo studies, mTORC1 inhibition and consequent autophagy activation in BAT can result in an increase in thermogenesis and the induction of lipolysis. Accordingly, it could be concluded that mTORC1 signaling and autophagy contribute to lipolysis regulation, and consequently, adipocyte development [53].

In addition to the role of autophagy in preadipocyte differentiation, it was supposed that its contribution in adipocyte maturation. In 3T3-L1 cells with *ATG5* and *ATG7* deficiency, C/EBPβ, which contributes to clonal expansion of preadipocyte and activates the transcription factors for adipocyte maturation, was downregulated [42]. The observed defective adipogenesis in animals with *ATG7* deletion in skeletal muscle suggest that changes in autophagic activity can play a role in the modulation of inter-organ crosstalk. Therefore, any primary/developmental disturbance in autophagy can influence the mass and homeostasis of adipose tissue [54].

Accordingly, autophagy inhibition could be considered a preventative measure against insulin resistance and obesity by increasing the development of BAT. However, the molecular mechanisms by which autophagy induces the trans differentiation of white adipose tissue (WAT) to BAT should be identified [48].

### Autophagy and mitochondrial dysfunction, their role in insulin resistance

Obesity and excessive nutrient consumption can lead to mitochondrial dysfunctions. The highest levels of mitochondrial dysfunctions in obesity were observed in adipose tissues, liver, and muscles [55]. Mitochondrial dysfunction enhances mitophagy, which is a quality control pathway for selective clearance of the excess mitochondria when mitochondria have accumulated during adipogenesis or have been damaged by oxidative stress [56–58]. It has been suggested that changes in the mitochondrial mass and function play a key role in the pathophysiology of insulin resistance [59]. Since in obesity, increased autophagy in adipose tissue results in elevated mitochondrial degradation, it is likely that elevated autophagy reduces the mitochondrial mass in adipocytes, increases the energy storage potential, and contributes to the pathogenesis of obesity [60]. Recent studies on autophagy in adipocytes have focused on the mitochondrial turnover pathways. Mice with adipocyte-specific inhibition of autophagy are resistant to diet-induced obesity and show improved insulin sensitivity [42, 43]. The findings suggest that autophagy has a critical function in adipocyte development through the clearance of mitochondria.

### Autophagy and ER stress in adipose tissue and their relationship with insulin resistance

Growing evidence indicates that ER stress and insulin resistance are potentially connected through autophagy [61–63]. Similarly, insulin resistance and autophagy-dependent apoptosis are likely to be caused by unresolved ER stress due to the activation of JNK phosphorylation by inositol-requiring kinase 1 (IRE1). Moreover, obesity induces ER stress in adipocytes, resulting in an increase in adiponectin levels, and the insulin receptor is misfolded and subsequently degraded through the autophagy-dependent pathway [62]. Zhou et al. showed that insulin receptors were significantly downregulated in 3T3-L1 adipocytes when were treated with ER stress inducers, as well as in the fat tissues of obese individuals [64]. It appears that autophagy has a role in the downregulation of insulin receptors in a condition of ER stress, such that the autophagy inhibitor 3-methyladenine (3-MA) blocked the ER-mediated degradation of insulin receptors [64]. Nonetheless, the ER stress-induced insulin signaling impairment in 3T3L1 adipocytes was not improved by blocking autophagy, indicating the dysfunction of served insulin receptors in the presence of ER stress [64]. It can be concluded that autophagy is stimulated in response to ER stress to clear toxic aggregates such as impaired IR.

Moreover, it was shown that the peripheral target tissues of insulin are the sites wherein autophagy is triggered by ER stress-induced insulin resistance [65]. On the account that some processes link autophagic disorders with ER stress responses and impaired insulin signaling through activating the JNK/IKK signaling pathways, ER stress, autophagy, and altered adipose tissue metabolism are all shown to be related to the development of insulin resistance [66].

Li et al. found that failed autophagy makes adipocytes susceptible to ER stress, which in turn leads to autophagy and insulin resistance in an IRE1–JNK pathway-dependent manner in mature adipocytes [62]. Overall, the downregulation of autophagy results in the growth of misfolded large molecules and aged/damaged organelles which would, in return, lead to the development of insulin resistance and ER stress. In general, the role of autophagy and its relation with ER stress and insulin resistance in adipose tissue are regarded as double-edged, and further analysis is needed to determine whether this role is protective or detrimental.

### Autophagy and adipose tissue inflammation and their effects on insulin resistance

Inflammation is one of the key processes leading to the development of insulin resistance in obesity [67]. It has been shown that the inflamed adipose tissue of obese individuals releases a variety of cytokines, such as IL-18, IL-6, IL-1, and TNF-α, thereby contributing to obesity-induced insulin resistance [38]. Based on previous animal and human studies, in adipose tissue adipocytes and macrophages, autophagy is upregulated as a result of obesity-associated inflammation. This pathway impacts the inflammatory status by regulating the gene expression and secretion of proinflammatory cytokines, such as IL-1, IL-6, and IL-8, which are involved in systemic glucose homeostasis and metabolic diseases. Therefore, it appears that autophagy is the result, not the cause, of obesity-induced inflammation in adipose tissue [38, 68]. In this regard, it has been demonstrated that in obese mice, global *ATG7* haplodeficiency leads to insulin resistance as well as the maturation of pro-IL-1β to IL-1β and increased inflammation [69]. Additionally, it has been reported that the deficiency of the autophagic protein ATG16L1 augmented IL-1β processing upon TLR4 stimulation. The exact mechanism by which the gene expression and subsequent secretion of inflammatory cytokines are enhanced after the inhibition of autophagy remains unknown. One possible mechanism may be that the inhibition of autophagy could increase IL-1 production in adipose tissue through the suppression of intracellular pro-IL-1 degradation, triggering the extracellular signal-regulated kinase (ERK)/nuclear factor-B pathway and the activation of inflammasome [70–72]. Moreover, mitochondrial autophagy is found to be correlated with insulin resistance and may also regulate the expression of inflammatory genes [73]. As an adipokine, leptin has been reported to decrease autophagy and consequently modulate adipose tissue inflammation. As shown in *ob*/*ob* mice, leptin prohibits autophagy through the downregulation of beclin-1 and ATG12, as well as the formation of the activating transcription factor (Atf) 4/ATG5 complex and reduces IĸB degradation by decreasing IL-18 and IL-1β in adipocytes, thus impacting ER stress and inflammation [74].

### The role of NLRP3 inflammasome in autophagy-related insulin resistance in adipocytes

Recent studies have shown that NLRP3 inflammasome disrupts metabolic regulation in adipose tissue by activating macrophage infiltration. However, the mechanism of NLRP3 activation and its role in adipocytes remain unclear. In a study by Javaid et al., TNF-α treatment induced NLRP3 expression and caspase-1 activity in adipocytes, partially through dysregulation of autophagy. High-fat diet regulates the expression levels of NLRP3 and caspase-1 in stromal vascular fractions and adipocytes. TNFα induces mitochondrial dysfunction, oxidative stress, and insulin resistance, which activate the adipose NLRP3 inflammasome without altering IL-1β. Active adipocyte NLRP3 inflammasome contributes to mitochondrial dysfunction and insulin resistance. Lipocalin 2, an NLRP3-dependent adipokine, mediated interactions between adipocytes and macrophages. NLRP3 mediates the deleterious effects of TNFα on mitochondria and glucose metabolism and regulates glucose uptake in adipose tissue. Lipocalin 2 from adipocytes could also activate macrophages, suggesting a role for adipose NLRP3 inflammasome activation in fat metabolism. This axis of TNFα–NLRP3–Lcn2 contributes to adipocyte–macrophage interaction and obesity-induced inflammation, creating a vicious cycle in adipose tissue [75].

### Targeting autophagy in obesity

Although decreasing caloric intake or absorption and bariatric surgery have been established as the main clinical approaches for the management of obesity, the long-term management of this condition seems to be challenging [76]. Preclinical evidence has proposed using autophagy modulators as a therapeutic approach for the treatment of obesity and metabolic diseases. However, it has not been proven whether the pharmacological targeting of autophagy has clinical efficacy in obesity [77].

In addition to pharmacological interferences, lifestyle modifications, such as exercise and caloric restriction, can counteract obesity and insulin resistance [78, 79]. The findings of previous studies have demonstrated that caloric restriction and exercise enhance autophagy in patients with obesity, and autophagy induction has a critical role in the beneficial impacts of these lifestyle modifications on obesity [80–83]. It has been shown that drugs that are utilized to reduce caloric intake by appetite inhibition, such as metformin, leptin, adiponectin, the agonists of glucagon-like peptide 1 (GLP1), liraglutide, and exenatide, serve direct regulatory functions in autophagy, however, the modulation of autophagy is mostly related to the inhibition of energy intake [84–89]. Moreover, incretin-based therapies, such as treatment with dipeptidyl peptidase 4 (DPP-4) inhibitors, which might impact autophagy, are considered as critical approaches for the treatment of obesity [90]. Based on the findings of animal studies, it has been demonstrated that similar to caloric restriction, ketogenic diets also have metabolic advantages, including resistance to obesity in mice, and increasing autophagy have a role in the beneficial impacts of ketogenic diets [91, 92]. Overall, the role of autophagy in adipose tissue development, and its activation in adipose tissue in obesity, propose that this pathway is an accepted modulator that could be targeted for therapeutic aims, since the inhibition of autophagy could be considered a strategy to ‘brown’ adipose tissues and enhance whole-body energy consumption to combat obesity. It is important to note that these findings should be interpreted with caution because many of the studies have been conducted on rodent models (especially mice) in which adipose tissue plasticity may be significantly higher than in humans and is based on species-specific neuroendocrinological mechanisms [60].

## Liver tissue

### The role of hepatic autophagy in the glucose homeostasis

Autophagy plays an important role in glucose homeostasis [93]. In this regard, hepatic autophagy is vital for the retention of energy balance, because one of the specific roles of the liver is to control homeostasis of glucose via retaining the concentration of blood glucose in the normal range and autophagy has an important part in this mechanism [94, 95]. During nutrient starvation, during short-term fasting, autophagy in the liver causes the production of glucose via the degradation of glycogen in the glycogenolysis pathway. But, in the prolonged fasting state, when the glycogen in the liver is depleted, hepatocytes produce glucose via the gluconeogenesis pathway [96–98]. Due to the role of autophagy in the liver in maintaining the blood glucose concentration, the results of a study on mice indicated that acute *ATG7* deletion in the fasting state was associated with apoptosis induction in the liver tissue, reduction in the liver size, depletion of the stores of glycogen and lipid in the liver, and severe hypoglycemia, suggesting that autophagy is essential for the endurance of fasting, maintaining blood glucose, and survival [99]. In addition, Yang et al. revealed that in mice that were genetically or dietary obese, following the expression of *ATG7* in the liver, insulin sensitivity was increased and glucose homeostasis was improved [100]. On the other hand, Liu et al. showed that in mice with hyperinsulinemia and insulin resistance induced by HFD, the expression of several important autophagy genes was inhibited. On the contrary, they showed that hepatic autophagy activity was enhanced in the group with insulin deficiency induced by streptozotocin (STZ), suggesting the suppression of autophagy in the state of hyperinsulinemia/insulin resistance [101].

Moreover, hepatic autophagy leads to the breakdown of proteins, and the resulting amino acids are converted to glucose in the gluconeogenesis pathway [102]. Generally, autophagy is inhibited by insulin and amino acids after food consumption, but in fasting states, it is stimulated through glucagon and the deficiency of amino acids [101]. Insulin inhibits hepatic autophagy via activating mTOR. The overabundance of nutrients is related to the enhanced activation of mTORC1 in the liver. Insulin can also downregulate numerous ATGs [103]. Unlike insulin, in fasting conditions, glucagon plays an important role in increasing blood glucose through the activation of glycogenolysis and gluconeogenesis and the inhibition of glycogenesis [104, 105]. In addition, glucagon activates autophagy by affecting the liver cells via increasing the number and size of autophagic vacuoles and upregulating lysosomal enzymes, acid phosphatase, and cathepsin D [106, 107]. It is suggested that glucagon via AMPK-mediated inhibition of mTORC1 activity, upgrades hepatic autophagy as well [108]. Nevertheless, the role of insulin is more important than glucagon in relation to controlling hepatic autophagy, because even at elevated plasma levels of glucagon, autophagy is induced only when blood insulin levels drop [109]. Taken together, hepatic autophagy is required to maintain the body's energy in a fasting state and insulin plays an important role in regulating the autophagy pathway.

### Pathophysiological role of autophagy in hepatic steatosis and NAFLD

NAFLD, as the most prevalent chronic liver disease, is closely linked to hepatic insulin resistance and is observed in approximately 25% of the population. It includes a spectrum of hepatic disorders from a simple intrahepatic accumulation of TG or steatosis to fibrosis, cirrhosis, and hepatocellular carcinoma at advanced stages [110–113]. Insulin resistance and fat accumulation in the liver are among the most important factors involved in the pathogenesis of NAFLD [114]. Hepatic steatosis is the first stage in NAFLD pathogenesis, which is usually benign, and is an abnormal condition caused by the over-accumulation of LDs containing TG in hepatocytes. Lipophagy prevents the development of hepatic steatosis by degrading LDs [115–118]. This disorder is the result of an imbalance in the de novo synthesis of fatty acids in the hepatocytes or an increase in the number of fatty acids entering the liver, leading to an enhanced TG synthesis, or reduced secretion of very-low-density lipoprotein (VLDL) from the liver [119]. As a result of insulin resistance in adipose tissue, lipolysis is enhanced and leads to increased levels of free fatty acids (FFAs) in the liver. Due to the inhibition of autophagy by hyperinsulinemia and the role of autophagy in the breakdown of TGs, hepatic steatosis is worsened [120].

Studies have shown that in mice with HFD, the deletion of the *Tfeb*, *ATG7*, and *ATG14* genes in hepatocytes, which encode vital autophagy regulators, as well as the deletion of the *Atg5* gene in myeloid or endothelial cells, is associated with increased lipid accumulation in the liver and the development of NAFLD [121–123]. Overall, the inhibition of autophagy may be considered one of the causes of NAFLD. In this regard, during insulin resistance, the resulting hyperinsulinemia leads to the inhibition of autophagy via the activation of mTOR and the reduction of FOXO1 [124]. Research has indicated that dyslipidemia and steatosis are related to the impairment of FOXO, which is a transcription factor and the main intracellular target of insulin [124, 125]. FOXOs activate autophagy by inducing the expression of autophagy genes [126]. For example, the overexpression of FOXO1 in the Hepa1c1c7 cells prevents the inhibition of major autophagy genes by insulin [101]. Because FOXOs stimulate the degradation of LDs by activating lipophagy, a defect in their function may lead to the development of NAFLD [127].

One of the important regulators of the autophagy pathway is the transcription factor EB (TFEB) [128]. This transcription factor controls the expression of genes implicated in the regulation of lipid metabolism in the liver (such as oxidation of fatty acids and synthesis of ketone bodies). It also activates autophagy by inducing related genes [129, 130]. In this regard, metformin, which is a drug known to enhance insulin sensitivity, reduced insulin resistance and hepatic steatosis in a NAFLD mouse model induced by HFD by promoting autophagy in a TFEB-dependent manner [131, 132]. In obese animals, the absence of TFEB leads to disruption in the catabolism of lipids, whereas its high expression has the opposite effect [133]. Therefore, the suppression of TFEB and consequently the inhibition of autophagy lead to the accumulation of lipids in hepatocytes and induce steatosis [128]. Generally, the stimulation of hepatic autophagy by activators may have therapeutic potential and minimize liver impairment and NAFLD.

### The role of autophagy in the regulation of hepatic lipid and lipoprotein metabolism and its importance in insulin resistance

The hepatic failure of lipid metabolism certainly contributes to the insulin resistance in this tissue and in the entire body tissues. Of all underlying mechanisms, autophagy impairments lead to the toxic accumulation of lipids, e.g., TG in the liver. In this regard, based on in vitro and in vivo studies, it is shown that the lack of some important autophagy genes such as *ATG7* and *ATG5* leads to the accumulation of TG levels in the liver; conversely, the overexpression of *ATG7* leads to a decrease in the hepatic TG [100, 122]. In addition to *ATG7* and *ATG5*, Xiong et al. revealed that ATG14, as an important factor in initiating autophagy, plays a critical function in the homeostasis of hepatic lipid metabolism. The overexpression of ATG14 in the liver of LTKO mice was shown to increase autophagy activity and decrease TG levels. Furthermore, the authors recognized one cluster and a single insulin response element (IRE) in the promoter of the *ATG14* gene which regulates the promoter activity of this gene [122]. Additionally, Li et al. showed that the dysregulation of miR199a-5p, as an important regulator in hepatic glucose and lipid metabolism, inhibited the expression of ATG14 and suppressed autophagy, which can lead to impaired hepatic insulin sensitivity and ultimately insulin resistance [134]. Based on the fact that the combined effects of mTOR and insulin lead to the regulation of autophagy, Liu et al. reported that insulin resistance and hyperinsulinemia were induced in mice fed an HFD, which in turn inhibited hepatic autophagy [101]. The results of the study by Ghareghani et al. revealed that HFD in mice leads to enhanced expression of lipogenic genes and the impairment of the autophagy pathway. Moreover, in the HepG2 cell line, autophagy suppression via chloroquine increased lipogenesis, and the induction of autophagy by rapamycin, as a specific inhibitor of mTOR, decreased lipogenesis [135, 136]. The activation of autophagy by rapamycin improved insulin resistance and hepatic steatosis in animal models of T2DM, which was associated with a reduction in the levels of TG and fatty acids in the liver tissue [137]. However, Di Ma et al. indicated that liver autophagy deficiency by FIP122 knockout did not develop steatosis in both control and HFD-fed mice. Decreased lipid accumulation in the liver of these animals can be due the entire reduced expression of genes implicated in lipid metabolism including lipogenesis [138].

FFAs are other factors involved in the induction of hepatic insulin resistance via several mechanisms, including PKC-δ, phosphorylation of Akt, IRS-1, insulin receptor, and de novo lipogenesis [139, 140]. In the context of the autophagy response to FFAs, Tan, et al. conducted a study on cells treated with palmitic acid, a saturated fatty acid, showing that the autophagic flux level was increased, whereas oleic acid, a monounsaturated fatty acid, had no such effect [141]. On the other hand, Mei et al. reported that oleic acid induced autophagy while palmitic acid decreased it in primary mouse hepatocytes and HepG2 cells [142]. However, both studies demonstrated that autophagy inhibition by drug or *ATG7* knockdown led to the obvious exacerbation of the toxic effects of palmitic acid and leads to further apoptosis in the palmitate-exposed cells, suggesting that autophagy acts as a protective mechanism against lipotoxicity. Fatty acid metabolites such as TG and DAG, involved in insulin resistance, trigger autophagy by which these are metabolized, raising the possibility that autophagy modulates liver insulin resistance by reducing TG stores [143].

In addition to TG, studies have documented that autophagy is implicated in lipogenesis, cholesterol efflux, ketogenesis, and oxidation of fatty acids [144]. For example, the accumulation of free cholesterol in hepatocytes leads to the dysfunction of lysosomes and the inhibition of hepatic autophagy; thus, the induction of cholesterol 7α-hydroxylase, as a rate-limiting enzyme in the biosynthesis of bile acids from cholesterol, leads to increased bile acid synthesis and decreased Akt/mTOR signaling, and ultimately activates autophagy [145–147].

Moreover, in relation to ketogenesis and autophagy, Saito et al. demonstrated that upon fasting, the loss of *ATG5* or *ATG7* in the liver disrupts the production of ketone bodies, indicating the importance of autophagy in the production of ketone bodies in fasting [144]. In addition, autophagy may control the level of VLDL particles, which are rich in TGs created in the liver [97]. The amount of VLDL and TG secretion from the liver depends on the availability of apolipoprotein B100 (apoB100), which is fundamentally produced via hepatocytes. Some studies have shown that the destruction of apoB100 is mediated by autophagy [148–150]. Andreo et al. showed that for the destruction of apoB100 in the insulin-stimulated hepatocytes, autophagy and class II PI3K are necessary. Additionally, the production of VLDL and apoB100 was excessive in those with insulin resistance [151]. Following the inhibition of autophagy by genetic and/or pharmacological factors, aberrant fat accumulation occurs in the liver, leading to insulin resistance [152]. The absence of lipophagy in the liver cells blocks the hydrolysis of TGs into FFAs and leads to steatosis and insulin resistance [53].

### Autophagy and insulin resistance in liver tissue

Under the feeding conditions, insulin is released from pancreatic β-cells, and by acting on muscle and adipose tissues, it increases energy storage in these tissues in the form of glycogen and TGs, respectively [153]. Following the binding of insulin to its receptors in hepatocytes, the PI3K/Akt pathway is stimulated. Akt subsequently activates mTORC1 and suppresses FOXO (which is involved in the initiation of autophagy), leading to the inhibition of autophagy by insulin [34, 154]. One of the mechanisms by which insulin can block autophagy is the suppression of the *vps34* gene, which is required for the initiation of autophagy, as well as *ATG12* and GABA type A receptor-associated protein like 1 (gabarapl1) genes, which are necessary for the formation of complex I and II of autophagy, respectively. The expression of these genes in hepatocytes is FOXO1-dependent [101]. The results of study on mice with insulin resistance and hyperinsulinemia induced via HFD indicated that the hepatic autophagy and the expression of several main autophagy genes were suppressed [101]. Interestingly, autophagy was found to affect insulin signaling such that hepatic autophagy impairment promoted obesity and induced insulin resistance [46]. The results of an in vitro and in vivo study revealed that *ATG7* suppression in the liver becomes an impairment in insulin sensitivity, and the recovery of *ATG7* gene expression in obese mice leads to increased hepatic insulin activity [100, 118]. Figure 3 shows the insulin signaling in liver tissue under physiological and pathological conditions (3.2.a and 3.2.b). With regard the intercalated relation between insulin signaling and autophagy, hence, the interaction should be further studied in the setting of insulin resistance.

### Autophagy and ER stress in liver tissue and their relationship with insulin resistance

Studies have revealed that following intense ER stress, the IRE1–JNK pathway is activated, which subsequently suppresses insulin receptor signaling in the liver cells [94, 155]. The secretion of fatty acids from adipocytes in obesity induces the unfolded protein response (UPR) pathway and by activating the JNK and inhibiting the expression of chaperone proteins and ER-associated degradation (ERAD), insulin resistance occurs in the liver [94]. Furthermore, some findings propose that the upregulation of tribbles-related protein 3 (TRB3), a pseudokinase that suppresses the activation of Akt, via the PERK pathway in diabesity could contribute to the induction of hepatic insulin resistance by ER stress [156, 157]. In addition to the ERAD, which degrades misfolded and unfolded proteins, autophagy is also induced by ER stress and UPR [158]. Autophagy induced by ER stress is considered a mechanism for the degradation of misfolded proteins that cannot be decomposed by ERAD [100]. ER stress induces autophagy via several mechanisms such as the IRE1–JNK pathway, ER Ca^2+^ leakage, the assembly of pre-autophagosome, the enhancing of AGT1 kinase activity, and the upregulation of *ATG* genes [159–163]. The results of a study on the rat model of NAFLD treated with an inhibitor of JNK showed that the expression levels of *ATG3* and *ATG5* were decreased, insulin resistance was reduced, and NAFLD was improved [164]. Nevertheless, it has been shown that the reduction of hepatic autophagy activity causes ER stress and insulin resistance [165]. In this regard, Yang et al. reported that in a starvation state, the subsequent imperfect hepatic autophagy in obesity and the suppression of AGT7 induce ER stress and insulin resistance, leading to the assembly of lipids in the liver [100]. Furthermore, it was reported that in the livers of murine models and patients with NAFLD, the autophagic pathway is impaired, which could be due to the fact that increased ER stress causes apoptosis [166]. Overall, the increase in ER stress and the decrease in autophagy activity may lead to impaired insulin signaling and insulin resistance in liver tissue.

### Autophagy, mitochondrial dysfunction, inflammation, and oxidative stress in the liver tissue and their links with insulin resistance

Mitochondrial dysfunction is a hallmark of chronic and acute liver disorders, such as NAFLD, alcoholic liver disease, and viral hepatitis [167, 168]. Mitochondrial dysfunction is also involved in the development of diabetes and insulin resistance, as mitochondria produce a high amount of ROS and oxidative stress plays an important role in insulin resistance [169–171]. One of the pathways by which ROS causes insulin resistance is JNK activation [172]. It was proven that FFAs promote mitochondrial dysfunction and accumulate in the liver, thus inducing hepatic insulin resistance [173]. Upon the accumulation of ROS and the elevation of oxidative stress as a result of increased proinflammatory factors and FFAs, the signaling pathways of various stress-sensitive serine/threonine kinases such as IKKβ, are activated and several targets of these kinases including IRS-1 and IRS-2 and the insulin receptor are phosphorylated at serine and threonine sites, resulting in decreased insulin function and insulin resistance [14, 174].

Autophagy is essential in limiting ROS generation by removing damaged mitochondria. On the other hand, autophagy is induced by ROS at several levels: In the initiation stage, through the modulation of AMPK and mTORC1 activity, in the nucleation phase, through the activation of protein kinase D and caveolin-1, and in the elongation step, through inducing the activation of *ATG4* [175]. Accordingly, the suppression of autophagy leads to increased production of ROS [176]. Furthermore, the inhibition of autophagy triggers the production of inflammatory cytokines IL-1β and TNF-α, by blocking NF-κB and inflammasome [177]. Studies have revealed that autophagy can negatively regulate the activation of the inflammasome via several mechanisms, including the elimination of damaged mitochondria, which resulted in a drop in ROS levels and the consequent inhibition of the inflammasome, p62-dependent breakdown of the inflammasome, and finally, the sequestration of pro-IL-1β [178]. It was reported that IL-1β disrupts insulin signaling and leads to insulin resistance in hepatocytes via decreasing IRS-1 expression [179]. In this regard, it was demonstrated that the alleviation of inflammation by removing Kupffer cells form the liver restored autophagy and improved insulin resistance in HFD-fed mice [180, 181]. Therefore, the reduction of liver macrophages can be a suitable strategy to increase hepatic autophagy and reduce liver inflammation, insulin resistance, and related metabolic diseases such as NAFLD and T2DM.

## Skeletal muscle tissue

Muscles are responsible for the body's movement and protection. However, continuous muscle contractions cause ROS production and damage muscle cell proteins. Thus, these cells need specific mechanisms to remove damaged proteins and organelles and supply amino acids from proteins [182]. Along with mechanisms such as proteasomal degradation, autophagy is one of the processes that can help to eliminate damaged proteins, prevent their accumulation, and convert them into amino acids.

Insulin, as the most important regulator of blood glucose, stimulates glucose uptake mainly via skeletal muscle. Muscle insulin resistance is considered an essential component of whole-body insulin resistance since skeletal muscle is responsible for about 60–70% of insulin-stimulated glucose disposal in the entire body [183]. Insulin-stimulated glucose absorption in skeletal muscle is significantly reduced under insulin-resistant conditions, allowing for the development of T2DM. However, autophagy can improve insulin resistance in skeletal muscle [184].

### Autophagy, insulin signaling, and insulin resistance in skeletal muscle tissue

Crosstalk between autophagy and insulin signaling is of particular significance. It was demonstrated that the inhibition of autophagy by chloroquine, bafilomycin, or the inactive ATG5 mutant diminished glucose uptake in the L6 skeletal muscle stimulated by insulin. In addition, considerable reductions were observed in the phosphorylation levels of Akt and IRS-1 [86]. Numerous studies have described the importance of autophagy in the insulin-resistant skeletal muscle in vivo. In HFD-fed mice and animal models of polycystic ovary syndrome (PCOS) with hyperinsulinemia and insulin resistance, autophagy reduced in skeletal muscle [86, 185]. However, six weeks of HFD feeding resulted in enhanced autophagy markers (LC3II and beclin), indicating that autophagy increased as a compensatory response to early insulin resistance [86]. Interestingly, genetic animal models with continuous hyperactive autophagy manifest more insulin sensitivity on the HFD challenge, as evidenced by increased phosphorylated Akt in the gastrocnemius skeletal muscle [93].

It is noted that insulin sensitizers, such as adiponectin, promote the autophagy pathway in skeletal muscle [86]. Exercise, which is known to improve insulin resistance, partly functions through autophagy [186]. In addition, Song et al. showed that dehydroepiandrosterone activates mTORC1 and inhibits autophagy, leading to insulin resistance in the skeletal muscle of mice with PCOS [185]. Shi et al. reported that dihydromyricetin, a flavonoid component of herbal medications, improves the skeletal muscle insulin sensitivity by inducing autophagy via the AMPK–PGC-1α–sirtuin 3 (Sirt3) signaling pathway [187]. The specific knockout of *ATG7* in skeletal muscle unexpectedly improved glucose uptake and insulin resistance following HFD feeding [54].

The skeletal muscle of T2DM patients with hyperinsulinemia exhibited a reduction in autophagy, which was demonstrated by the reduced expression of *SQSTM1/p62*, *ATG14*, *GABARAPL1*, RB1 inducible coiled-coil 1* (RB1CC1)/* family-interacting protein of 200 kDa* (FIP200)*, and WD repeat domain phosphoinositide-interacting protein 1 (*WIPI1*)genes, decreased levels of SQSTM1/p62, LC3BII, and ATG5 proteins, as well as the elevated phosphorylation of FOXO3a [188]. These data indicate that insulin resistance is associated with autophagy inhibition in muscles while increased insulin sensitivity had inverse effects [189]. Conversely, Kruse et al. did not find any changes in the mRNA and protein levels of the autophagic markers of the skeletal muscle in T2DM patients, suggesting that markers of autophagy are probably adapted to hyperglycemia in skeletal muscle in T2DM patients [189]. Regarding the in vivo impact of insulin on autophagy, it is noted that the glycemic status, as a crucial factor, as along with insulin affect autophagy in patients with diabetes [190]. Insulin infusion led to a decreased LC3BII/I ratio in obese and lean individuals, but not in diabetic patients, indicating that insulin is capable of inhibiting autophagy in conditions other than diabetes [189]. In this context, the results of the study by Lv et al. are noteworthy. They investigated autophagy alterations in hyperglycemic conditions with or without hyperinsulinemia, by using both animal models of hyperglycemia, i.e., hyperglycemia by glucose infusion and hyperglycemia induced by STZ. In STZ-treated rats, which present hyperglycemia and low insulin, autophagy proteins (LC3, ATG5, ATG12, and BECN1) were significantly elevated in the gastrocnemius muscle, which contributed to the increase of p-FOXO3a and the subsequent repression of mTOR. In contrast, in animals with glucose infusion hyperglycemia, which present hyperinsulinemia, autophagy markers, including LC3-II/I, ATG7, and BECN1 decreased in gastrocnemius muscle, indicating that insulin in the lack of insulin resistance can suppress autophagy in skeletal muscle [191]. It appears that the status of insulin and glycemia, as well as insulin sensitivity, altogether create a unified response that determines the stimulation or inhibition of the autophagy process.

Furthermore, the glucagon level, which is elevated in the presence of high insulin and insulin resistance state, is another main factor that regulates autophagy [192]. The effects of glucagon on the autophagy pathway are the opposite of insulin’s effects, i.e., glucagon activates autophagy. It has been found that chronic exposure to insulin diminishes the stimulating effects of glucagon on autophagy in hepatocytes [101], indicating that insulin is more important than glucagon in autophagy regulation. However, more research is required to clarify the in vivo effects of different variables on skeletal muscle autophagy. Overall, the autophagy function is balanced under normal conditions, whereas it is inhibited in the presence of insulin. Nevertheless, it may be increased as a compensatory response to maintain homeostasis. Figure 3 shows insulin signaling in modules under physiological and pathological conditions (3.3.a and 3.3.b).

### Autophagy and metabolism of glucose and lipids in skeletal muscle tissue

Insulin resistance is associated with elevated levels of glucose, circulating fatty acids, and TG, as well as lipid accumulation, which is linked to metabolism abnormalities in the skeletal muscle [193]. It is well established that an HFD results in the accumulation of lipid derivatives, such as triacylglycerol, diacylglycerol, and ceramide, in skeletal muscle, leading to insulin resistance [194]. Several studies support the role of autophagy in lipid metabolism, suggesting that it is impaired in pathological conditions such as insulin resistance [86, 185, 195]. It was demonstrated that p62, an autophagy receptor targeting cargo to autophagosomes, is found on LDs, which indicates that lipophagy participates in the removal of TG from muscle cells such that the treatment of fatty acid-exposed muscle cells with rapamycin (an autophagy activator) reduced lipid accumulation while bafilomycin (an autophagy inhibitor) showed the opposite effect [196]. On the other hand, lipids can modulate the autophagy process in skeletal muscle cells. It was demonstrated that HFD globally reduces the expression of autophagy genes in the soleus (SOL) muscle [197]. Palmitate, a saturated fatty acid with high concentrations in circulation, and known to promote insulin resistance in skeletal muscle, impaired the autophagic flux in C2C12 cells [195]. These data suggest that autophagy impairment contribute to lipid accumulation and thereby insulin resistance in muscles. In contrast, Morales-Scholz concluded that autophagy is neither implicated in lipid accumulation nor in the development of insulin resistance in skeletal muscle. In this study, feeding rats with a high saturated-fat diet (HSFD) for 16 weeks did not change the autophagic markers in their skeletal muscle, but autophagy defects were seen in the liver [198]. The difference between oxidative muscles and glycolytic muscles in autophagy is of particular significance. While autophagic proteins (LC3-I, LC3-II, and SQSTM1) were higher in the oxidative muscle compared to the glycolytic muscle, autophagic flux was low in the oxidative muscle [199]. In female mice fed an HFD, p62 and ATG12-5 accumulation was induced in the oxidative muscle (SOL) but not in the glycolytic muscle (plantaris) [200]. It is noted that HFD induces the accumulation of diacylglycerols and ceramides in oxidative muscles SOL and extensor digitorum longus (EDL) but not in the glycolytic muscle epitrochlearis (Epit) [201]. It appears that the importance of autophagy with regard to lipid accumulation in muscles depends on the muscle type.

In addition, it has been shown that autophagy activity is related to glucose homeostasis. It was found that the glycogen stores of the skeletal muscle were mobilized through the autophagy degradation pathway in newborn rats [109]. The hexokinase (HK) enzyme, which catalyzes the first reaction of glycolysis, exerts a modulatory effect on autophagy [202]. Roberts et al. demonstrated that HKII suppresses the autophagy inhibitor mTORC1, thereby activating autophagy [203]. In C2C12 cells under the condition of low glucose concentrations, HKII was upregulated, which is associated with the induction of autophagy [204]. It appears that HKII, as a regulatory molecule, plays a crucial role in the metabolic switch from glycolysis to autophagy during glucose starvation. Given that HKII is regulated by insulin, its possible downregulation during insulin resistance leads to impairments in the autophagy pathway.

It should be noted that skeletal muscle autophagy may be altered in response to pathological conditions manifesting metabolic syndrome. For example, in NAFLD, myocellular autophagy is enhanced as a metabolic response to hyperammonemia [205], or in animals with hypertension, autophagy markers, including LC3I, ATG7, and lysosome-associated membrane protein 2 (LAMP2), are increased in skeletal muscle [206]. It can be concluded that the metabolism of muscle cells is influenced by insulin resistance, which is associated with autophagy dysregulation [205, 206].

### The role of myokines in autophagy-related insulin resistance

Myokines are peptides or cytokines that are synthesized and secreted by skeletal muscle, with autocrine, paracrine, and endocrine activities. They include myonectin, myostatin, apelin, fibroblast growth factor 21 (FGF21), brain-derived neurotrophic factor (BDNF), irisin, IL-15, IL-6, and other similar factors [207]. Myokines are potentially involved in regulating metabolism, autophagy, and insulin resistance as well as crosstalk between skeletal muscle and other tissues [208].

Myostatin is known as a negative regulator of skeletal muscle mass. In addition, it plays a role in the pathogenesis of insulin resistance. Its circulating levels are increased in obese patients with insulin resistance, and it has a negative correlation with adiponectin levels and insulin sensitivity indices [209]. In C2C12 skeletal muscle cells, it was found that myostatin exerted deleterious effects on the factors involved in the insulin signaling pathway, including IRS-1, glycogen synthase kinase-3 (GSK3), Akt, GLUT4, AMPK, and PGC1α [210]. Furthermore, it was reported that myostatin potentiates the autophagy process in C2C12 cells, accounting for muscle mass loss and muscle atrophy in these cells [211]. Studies have shown that myostatin inhibition by gene silencing [212], anti-myostatin oligomers [213], or by anti-myostatin antibodies [214] improves insulin resistance. It appears that the improvement of insulin sensitivity by myostatin suppression is due to the increased muscle mass and insulin signaling reinforcement rather than autophagy regulation.

Irisin is primarily produced by the skeletal muscle. Both autophagy and irisin increase in skeletal muscle following exercise and it seems that both mediate the beneficial effects of exercise on skeletal muscle insulin resistance [215]. In insulin-resistant C2C12 skeletal muscle cells, it was found that irisin could induce the autophagy process through the p38 MAPK/PGC-1α axis, improving insulin signaling and glucose uptake [216].

Several studies have reported that CTRP15 is related to insulin resistance in patients with metabolic syndrome [217], coronary artery disease [218], T2DM [219], and PCOS [220]. Its serum levels significantly increased in individuals during exercise [221]. It has been demonstrated that CTRP15 negatively regulates liver autophagy via Akt/mTOR signaling, as evidenced by reduced *Atg* expression and p62 degradation [222].

IL-15 and IL-6 are cytokines secreted by skeletal muscle cells, addition to the immune cells during immune responses, and are involved in the pathogenesis of autoimmune diseases and insulin resistance [223]. IL-15 has been shown to regulate skeletal muscle mass by suppressing the ubiquitin proteolytic process. In addition, it directly stimulates glucose uptake in muscle cells [224, 225]. High levels of IL-15 contribute to the improvement of insulin resistance in postmenopausal women with metabolic syndrome [226]. IL-15 triggers autophagy in the immune cells, mediating homeostasis and the survival of these cells [227, 228]. IL-6 is a pleiotropic cytokine with respect to insulin resistance; while numerous studies have indicated its association with insulin resistance [226–229], others have reported its insulin-sensitizing effects [230, 231]. It has been found that IL-6 inhibits starvation-stimulated autophagy in pro-monocytic U937 cells [184]. Nevertheless, it is still unclear whether IL-15 and IL-6 trigger autophagy in order to regulate insulin signaling in skeletal muscle cells.

Overall, myokines provide crosstalk between skeletal muscle and other tissues and serve functions in all tissues. The available data are not sufficient to fully describe their contribution to autophagy-related insulin resistance, and since skeletal muscle mass is crucial in insulin sensitivity and a high autophagy rate leads to muscle loss, it should be considered to gain a correct understanding of the role of myokines in autophagy-related insulin resistance.

### Autophagy, mitochondrial dysfunction, ER stress, and oxidative stress in skeletal muscle tissue and their links with insulin resistance

A growing body of evidence links mitochondrial dysfunction and oxidative stress to insulin resistance [229]. Particularly, in the muscle, it has been shown that elevated ROS levels stimulate the p38 mitogen-activated protein kinase (MAPK)/JNK/ERK pathway, which is involved in insulin resistance. Furthermore, increased levels of ROS activate mitophagy in the skeletal muscle such that the treatment of myotubes with H_2_O_2_ augments the levels of phosphorylated ULK1 and LC3 proteins [230]. Autophagy can mutually modulate mitochondria function and oxidative stress. In the skeletal muscle of *ATG7*-deficient mice, the protein carbonyl level, as a biomarker of oxidative stress, was increased and the ATP content and cytochrome oxidase activity were reduced, suggesting that autophagy deficiency leads to oxidative stress and mitochondrial impairments in the muscle, which is associated with reduced insulin resistance [54]. *ATG7* deficiency in the muscle is associated with the induction of FGF21. FGF21 is a mitokine that increases browning WAT and fatty acid oxidation, thereby improving insulin sensitivity [54]. Kim et al. reported that mitochondrial dysfunction in the muscle with *ATG7* deficiency improves insulin resistance. This finding is in contrast with the results of previous studies, which stated that mitochondrial dysfunction and autophagy impairment exacerbate insulin resistance [47, 184, 185]. It appears that the outcomes of autophagy deficiency depend on (1) where it occurs and (2) the component of the autophagy mechanism that is disrupted.

Considering the importance of muscle mass in whole-body responses to insulin, individuals who are prone to muscle mass loss are more likely to develop insulin resistance [231]. Oxidative stress in muscles initiates the process of muscle atrophy via activating autophagy, thereby decreased insulin resistance [232]. For example, neuronal nitric oxide synthase (nNOS) reduces muscle mass by activating oxidative stress and increases the expression of atrophy-related ubiquitin ligase genes (atrogin 1 and *MuRF1*) [233]. Moreover, MAPK induces autophagy during oxidative stress [234]. The formation of autophagosomes in myofibers is associated with atrophy and muscular dystrophy [235]. In addition, it has been indicated that the overexpression of PGC-1α (a mitochondrial biogenesis inducer) in skeletal muscle improves age-associated insulin resistance and reduces age-induced autophagy, thereby preventing muscle mass loss during aging [238]. However, the muscle-specific knockouts of autophagy genes, including *Atg5*^−/−^ and *Atg*7^−/−^, lead to muscle atrophy, presenting a myopathy-like phenotype [236, 237]. All the data demonstrated that proper muscle function requires the precise regulation of autophagy.

ER stress was observed in skeletal muscle with insulin resistance. It has been shown that ER stress disrupts insulin signaling in skeletal muscle and therefore reduces glucose uptake, which is mediated by tribbles-like protein 3 (TRBP3) [156]. ER stress is also known as an autophagic inducer, since the three main arms of ER stress, i.e., PERK, IRE1α, and ATF6α, trigger autophagy by activating JNK, X-box binding protein 1 (XBP1), and ATF4 [238]. It has been found that the treatment of C2C12 skeletal muscle cells with an ER stress inducer augments the conversion of LC3-I to LC3-II and decreases p62. In these cells, PKC-ϴ is demonstrated to mediate the ER stress-induced effects on autophagy [239]. The activation and membrane localization of PKC-ϴ might contribute to identifying membrane vesicles that form autophagosomes. Another candidate molecule that links ER stress to autophagy is skeletal muscle- and kidney-enriched inositol polyphosphate 5-phosphatase (SKIP). This phosphatase, which is upregulated under ER stress, negatively regulates insulin signaling by dephosphorylating PI3, and therefore, stimulating autophagy [240]. In general, ER stress is followed by autophagy activation, as a protective mechanism to remove misfolded proteins and thus relieve ER stress [238], providing cell survival. Additionally, the alleviation of ER stress is one of the main mechanisms by which autophagy lowers insulin resistance [93]. It was found that the genetic hyperactivation of autophagy improved HFD-induced insulin resistance in insulin-responsive tissues such as skeletal muscle, which was attributed to relieved ER stress, as evidenced by reduced levels of ER stress markers CHOP and ATF-6 factor in the skeletal muscle, liver, and adipose tissue [93]. In support of these findings, it was shown that ER stress inducers reversed the beneficial effects of autophagy hyperactivation in the aforementioned tissues [93].

However, autophagy acts as a defense response against ER stress, and depending on the degree of insulin resistance, this process is affected in different ways [61]. It should be noted that autophagy dysregulation may contribute to ER stress-mediated insulin resistance. In this regard, it is demonstrated that autophagy inducers (rapamycin or adiponectin) relieved ER stress and insulin resistance in skeletal muscle L6 cells while autophagy suppression had the opposite effect [241]. Therefore, it is concluded that under insulin resistance conditions, dysfunction of autophagy makes cells more vulnerable to ER stress.

Skeletal muscle insulin resistance is linked to inflammation, and the increase of inflammation enhances skeletal muscle insulin resistance [242]. IL-1, TNF-α, C-reactive protein (CRP), and monocyte chemoattractant protein-1 (MCP-1) are associated with skeletal muscle insulin resistance [243]. Insulin signaling is mainly disrupted by inflammation, ER stress, oxidative stress, the activation of the JNK and NF-кB pathways, and the increase of skeletal muscle insulin resistance. The JNK pathway and TNF-α disrupt insulin signaling by phosphorylating IRS-1 on the serine residue [244, 245]. IL-1 is demonstrated to activate the IKK/NF-кB pathway in the skeletal muscle, decrease IRS-1 activity, and enhance skeletal muscle insulin resistance. Moreover, IL-6 increases the expression of the suppressors of cytokine signaling (SOCS) 1 and SOCS3, leading to the degradation of IRS-1 and the development of insulin resistance [246]. Inflammation can also increase nitric oxide production, inhibit the PI3K-Akt pathway, and promote insulin resistance [244]. Overall, it seems that autophagy, mitochondrial dysfunction, ER stress, inflammation, and oxidative stress in the skeletal muscle are closely related to each other as a network and can all be affected by insulin resistance.

### Autophagy abnormality in β-cells under metabolic disorders

Metabolic disorders, such as obesity and diabetes, affect β-cell function through various mechanisms, including the dysregulation of autophagy. In these conditions, excess nutrients compel β-cells to secrete more insulin to maintain normal glucose levels. This increased demand on β-cells overloads the ER, leading to ER stress, poly-ubiquitinated protein aggregates, incomplete insulin processing, and/or irregularities in insulin secretion [247]. It has been established that blocking autophagy through chemicals or short hairpin RNAs (shRNAs) enhances the death of β-cells under ER stress, while treatment with rapamycin, an autophagy inducer, increases the viability of β-cells in the presence of ER stress, which demonstrates the protective role of autophagy against ER stress in β-cells [248]. Furthermore, evidence indicates that an excess of lipids and the resulting lipotoxicity, which is accompanied by metabolic abnormalities, significantly contribute to the impairment of β-cells. The expression of genes involved in lipid metabolism, oxidative stress, and apoptosis exhibits an abnormal pattern within the β-cells of individuals with type 2 diabetes [249]. In these cells, the accumulation of lipid droplets is linked to the downregulation of transcription factor EB (TFEB), which is a crucial modulator of autophagy. This, in turn, led to a decrease in the levels of the lysosomal biomarker LAMP2 [249]. Hyperglycemia is another primary factor that affects β-cells in metabolic disorders. It seems that chronic hyperglycemia or glucotoxicity affects lysosomal degradation in islets [250]. Lipid overload and high glucose levels reduce the expression levels of TFEB, LAMP1, and LC3, as well as p62 aggregation in INS‐1 cells, indicating that autophagy is dysregulated by hyperlipidemia and hyperglycemia [249]. Glucotoxicity also leads to increased production of ROS and mitochondrial abnormalities in β-cells [251]. It has been demonstrated that ROS, including H_2_O_2_, can induce autophagy as a protective mechanism [252]. In this context, when β-cells are exposed to amyloid polypeptides, ROS triggers an autophagy response to mitigate cellular injury. Conversely, the inhibition of autophagy worsens oxidative stress and β-cell damage [253]. Human islet amyloid polypeptide (hIAPP) is another key element involved in the pathogenesis of β-cell dysfunction in patients with type 2 diabetes [254]. As autophagy is responsible for clearing hIAPP, a deficiency in autophagy results in the accumulation of hIAPP, subsequently contributing to the development of diabetes [255]. It was found that the inhibition of autophagy exacerbates oxidative stress and β-cell damage induced by amyloid polypeptides [253]. Overall, reduced autophagy function resulting from genetic predisposition, aging, and obesity may contribute to β-cell impairment and diabetes. Therefore, the modulation of pancreatic β-cell autophagy is a promising approach for addressing diabetes related to hyperlipidemia, hyperglycemia, and the accumulation of islet amyloids.

## Complications

### Obesity and inflammation

Sarcopenic obesity has become a notable global health concern. The loss of muscle mass and fat accumulation in obesity causes an imbalance in myokines and adipokines, resulting in the development of insulin resistance and chronic inflammation [256]. Obesity, which is known as a condition of insulin resistance, is associated with chronic inflammation. It has been demonstrated that inflammatory factors, including IL-1, TNF-α, CRP, and MCP-1, are linked to tissue insulin resistance, and this relationship is mediated by the JNK and NF-кB pathways [256]. It was found that IL-1 and TNF-α disrupt insulin signaling by phosphorylating IRS-1 on the serine residue [257, 258]. Furthermore, in the progression of obesity, the imbalance between food intake and energy expenditure leads to autophagy suppression owing to the activation of mTOR signaling [77]. It seems that inflammatory factors are implicated in the autophagy dysfunction observed in insulin resistance-associated obesity. Consistent with this finding, it has been indicated that inflammatory agents, such as lipopolysaccharides, induce autophagy dysregulation, as evidenced by the decreased levels of ATG5, ATG6, and beclin 1, as well as increased p62 levels in macrophages [63, 259]. In addition, autophagy was impaired in the macrophages of HFD-induced obese animal models presenting chronic inflammation and insulin resistance [63]. In contrast, in the adipose tissue of obese individuals with moderate degrees of inflammation, an upregulation of autophagy markers was observed [257], which might be part of the defense response. Autophagy and inflammatory events are shown to be mutually linked. It is well established that autophagy influences IL-1β production via the inflammasome complex in neutrophils [258] and macrophages [259]. IL-1β is upregulated in macrophages that are Atg16L1-deficient [259]. Additionally, rapamycin (an autophagy activator) attenuates inflammatory cytokines, thereby improving insulin resistance [137]. Taken together, these findings demonstrate the interactions of autophagy and inflammatory agents and highlight the importance of autophagy in inflammation-regulated insulin resistance.

### Nephropathy

Nephropathy is characterized by a progressive rise in albuminuria, blood pressure, hyperfiltration, and podocyte loss as well as a decline in the glomerular filtration rate [260]. Nephropathy represents the disruption of the glomerular filtration barrier (GFB) [261, 262].

Autophagy maintains glomerulus and tubule homeostasis by recycling macromolecules and damaged cytoplasmic components and serves as a renoprotective mechanism for renal cells against stressful conditions [263, 264]. Recent evidence suggests that an imbalanced autophagic process is implicated in glomerular dysfunction and tubulointerstitial pathologies in the kidneys under insulin resistance conditions [265, 266]. Podocytes have a high level of autophagy activity even under normal conditions, implying that they require a basal level of autophagy function to maintain the integrity, normal structure, and function of the kidney [260]. Recent research has revealed that alterations in autophagy contribute to podocyte detachment with consequent albuminuria and a decline in filtration capacity. For example, the podocyte-specific deletion of *Atg* genes leads to podocyte loss and accelerates glomerulopathy [267]. Numerous studies have reported that the autophagy response in podocytes is lower in insulin resistance conditions [266, 268]. High glucose concentrations in cultured podocytes lead to molecular changes including the prevention of the normal autophagy response by restricting the expression level of the beclin-1, LC3, and Atg12-5 complex. This dysregulation of basal autophagy diminishes the filtration barrier function of podocytes [260]. Several investigations have indicated that the autophagy response is declined in the podocytes of insulin-resistant patients and animal models. In particular, autophagy insufficiency was observed in the kidney biopsy of patients with diabetic nephropathy and the animal models of insulin resistance with renal accumulation of p62/SQSTM1 and decreased levels of lysosome-associated membrane protein type 2A (LAMP-2A), which was accompanied by dysregulated autophagy function, enhanced lysosome dysfunction, and apoptosis [267].

### Retinopathy

The diagnosis of retinopathy is made on the basis of impaired retinal neuronal function, apoptosis of neuronal cells, especially amacrine cells, retinal ganglion cells, and photoreceptors, the thinning of the nerve fiber layer, pericyte loss, the breakdown of the blood–retinal barrier (BRB), the activation of Müller cells or reactive gliosis, and the overexpression of vascular endothelial growth factor (VEGF) and proinflammatory cytokines [269, 270]. Retinopathy is associated with decreased contrast sensitivity, slow adaptive response to dark, constricted visual fields, and finally, visual dysfunction [271].

There is increasing evidence that high basal autophagy is particularly maintained in the retinal pigment epithelium (RPE) and photoreceptor cells with normal retinal morphology and visual performance [269]. This baseline autophagy response reduces RPE and photoreceptor death as well as neural degeneration through the increased recycling of toxic rhodopsin. An inadequate autophagy flux leads to light-induced retinal degeneration. It has been reported that retinal autophagy is upregulated in individuals and mice with insulin resistance [270]. Autophagy enables retinal cells to counteract intracellular stress; however, in insulin resistance conditions, the autophagy machinery is a double-edged sword. Autophagic response in retinal cells may contribute to cell survival or the disruption of cellular homeostasis and cell death, depending on the level of stress and rBRB leakage. Autophagy activation in insulin resistance is a protective response against cell death in moderate stress, but it is an adverse response in excessive stress and contributes to cell death [272].

Müller cells or retinal glial cells (RGCs) cover all the retinal layers. The preliminary induction of autophagy prevented RGC death, but hyperglycemia-induced uncontrolled excessive autophagic response increased p62/SQTSM1 levels and led to an accelerated apoptotic rate and substantial VEGF formation [272]. The inhibition of the autophagy machinery by 3-methyladenine (3-MA) and chloroquine aggravated RGC apoptosis in a hyperglycemia environment while treatment with rapamycin improved the autophagic cargo degradation, and subsequently, promoted RGC viability [273].

Treatment of RPE cells with a high concentration of glucose accelerated ROS production, increased cell toxicity, reduced cell viability, and enhanced autophagic processes. These adverse impacts were eliminated by ROS scavengers and exacerbated by autophagy inhibitors in a dose- and time-dependent manner, indicating the protective role of autophagy in these cells [274]. In early retinopathy, autophagy activation attenuates pericyte loss as well as BRB disruption and enhances cell survival [269]. Pericytes are highly specialized cells that cover the retinal capillaries. As retinopathy develops and due to BRB leakage, autophagic death occurs. Pericyte loss leads to the obstruction of capillaries and microaneurysms [269].

### Cardiomyopathy and vascular dysfunction

Cardiomyopathy is characterized by impaired cardiomyocyte contractility and performance [275]. There is evidence indicating that autophagy dysregulation is involved in cardiomyopathy; for example, cardiac ablation of the *ATG5* gene leads to an increased incidence of cardiac dysfunction [276]. However, the cardiac autophagy activity in insulin resistance conditions has not been clearly described, and contradictory roles have been reported for cardiac autophagy in type 1 and type 2 diabetic animals [277]. Autophagic flux is enhanced in the heart of STZ-treated mice, as evidenced by the abundance of autophagic vacuoles or autophagosomes, the LC3-II/I ratio, and SQSTM1/p62 [277]. ATP deficiency or the accumulation of AMP in type 1 diabetic heart promotes AMPK activity in order to initiate autophagy, which results in cardiac failure [277]. Furthermore, treatment with chloroquine, an autophagy inhibitor, worsened cardiac diastolic and systolic performances in STZ-induced diabetic mice [277]. However, in db/db mice, as a model of T2DM, autophagy is disrupted in the step of autophagosome fusion with lysosome in heart tissue [277]. Moreover, the cardiac autophagy process was inhibited in obesity and HFD-induced insulin resistance, which was associated with increased levels of p62 protein and decreased levels of LC3II [278]. Several studies have revealed that the hyperactivation of autophagy in insulin resistance leads to cardiac cell degeneration and cytotoxicity [279]. Autophagy not only plays a crucial role in aberrant cardiac function but also contributes to vascular dysfunction and atherosclerosis pathogenesis. It has been shown that aortic dissection is exacerbated by ATG5 deficiency in vascular smooth muscle cells (VSMCs), which also prevents autophagosome formation and increases cell death [280]. Neointima development, atherogenesis, and accelerated VSMC senescence are all linked to defective autophagy in VSMCs [280]. It has been reported that chaperon-mediated autophagy is stimulated during initial pathogenic events, but it decreases upon atherosclerosis progression [281]. These data imply that basal autophagy functions as a protective mechanism to preserve the homeostasis of VSMCs and endothelial cells and prevents plaque formation, while excessive autophagy may exert the opposite effects.

### Infertility

Autophagy plays a critical role in female reproductive function by regulating folliculogenesis, the survival of germ cells, implantation, the maintenance of placental physiology, pregnancy, and anovulation [282]. A larger number of autophagosomes were detected shortly after fertilization, indicating the necessity of autophagy after fertilization, which removes unnecessary cellular proteins and supplies the acquired amino acids for embryonic progression [283]. It has been reported that the expression of autophagy-related genes is downregulated in the endometrium of women with PCOS and insulin resistance, and metformin (an insulin-sensitizing drug) counteracts these alterations [284]. Autophagy dysregulation was also reported in primary human granulosa cells with insulin resistance [285]. On the contrary, autophagy was upregulated in the ovarian tissue of PCOS rats and humans [135]. It has been reported that the overactivation of autophagy contributes to the impairment of HFD-related spermatogenesis since treatment with chloroquine, an autophagy inhibitor, ameliorates spermatogenesis defects in animals [286]. Moreover, excessive autophagy was observed in semen samples obtained from obese individuals [286]. Overall, although autophagy function is not necessary for the reproductive system to work correctly, it may play a different role in pathological conditions, implying the dual role of autophagy.

## Targeting autophagy in the treatment of diabetes

Table 1 indicates the Food and Drug Administration (FDA)-approved medications that possess a recognized indication on metabolic processes and have the potential to impact the autophagic pathway. Given that autophagy activity is inhibited in diabetic patients, new treatment strategies concentrating on autophagy restoration have been developed. To treat diabetic kidney disease (DKD), antioxidants can be used to combat oxidative stress, or chemical chaperons can be employed to lower ER stress and restore autophagy activity [287]. Reduced autophagy may be implicated in the development of DN when AMPK activation is reduced. Therefore, AMPK activation could be a target for reactivating autophagy. Reduced AMPK activity, and consequently, reduced cardiac autophagy are key factors in the progression of diabetic cardiomyopathy [84, 288]. In this regard, certain drugs, such as metformin and 5-aminoimidazole-4-carboxamide ribonucleotide, may alleviate DN by restoring autophagy. Furthermore, Zhou et al. showed that tea polyphenols improved rat heart function in diabetic cardiomyopathy and might modify autophagy levels to enhance lipid and glucose metabolism in diabetes [289].

**Table 1:** FDA-approved medicines with established indications on metabolism that could modulate autophagy

Medicine	Target	Mechanism of action	Tissue	References
Rapamycin	↓ mTOR signaling	- Inhibits mTOR	Adipose and liver	[137, 304]
Imatinib	- Inhibits BCR-ABL/PDGFR/KIT - Increases ROS	- Lowers mitochondrial membrane potential - Inhibits oxygen consumption and glycolysis - Promotes WAT browning - Disrupts the PI3K/Akt/FOXO4/ATF5/mTOR pathway	–	[296, 297]
Pioglitazone	Activates the PPAR and NAF-1	- Decrease apoptosis and inflammation	Adipose and liver	[298]
Liraglutide	↓ mTOR signaling	- Improves hepatic lipase activity - Decreases hepatic lipid content - Reduces glucose levels	Liver	[300]
Metformin	↑ AMPK	- Restoring SIRT1-mediated autophagy induction	Liver	[284, 293]
Resveratrol	↓ AKT1–mTOR pathway ↑ AMPK	- Activates sirtuin - Inhibits ribosomal protein S6 kinase	Adipose and liver	[305, 306]
Trehalose	Attenuates the phosphorylated levels of Foxo1 and p38 MAPK	- Blocks glucose transport	Muscle and liver	[294, 295]
Bortezomib	↑ AMPK	- Reduces the activation of the mTOR pathway, ER stress, and inflammation	Adipocytes	[299]
Sitagliptin	↑ AMPK	- Decrease TG, LDL-c, and oxidative stress - Suppress the mTOR signaling pathway	–	[118, 302]
Ezetimibe	- Inhibits cholesterol absorption	- Inhibits steatohepatitis via AMPK–TFEB- mediated activation of autophagy - Inhibits NLRP3 inflammasome	Liver	[307]

*AMPK* AMP-activated protein kinase, *ATF5* activating transcription factor 5, *ER* endoplasmic reticulum, *FOXO1* Forkhead Box O1, *LDL* low-density lipoprotein, *mTOR* mammalian target of rapamycin, *NAF-1* nutrient-deprivation autophagy factor-1, *PDGFR* platelet-derived growth factor receptor, *PI3K* phosphoinositide 3-kinase, *PPAR* peroxisome proliferator-activated receptors, *TG* triacylglycerol, *WAT* white adipose tissue. ↑ indicates "increase" and ↓ indicates "decrease"

Metformin, adiponectin, leptin, the GLP1 agonists liraglutide and exenatide, oxyntomodulin (which is most likely a GLP1 agonist), the cannabinoid receptor antagonist rimonabant, and the histamine receptor agonist betahistine all have direct regulatory effects on autophagy [74, 85, 290]. Modulation of autophagy, on the other hand, is closely linked to a reduction in calorie intake. Furthermore, thiazolidinedione pioglitazone, an insulin sensitizer that acts in part by activating the PPAR, has been found to enhance autophagy [291, 292].

Other well-known autophagy promoters including trehalose, rapamycin, and imatinib are also employed in clinical trials to increase insulin sensitivity and cell function [293–297]. Imatinib, a tyrosine kinase inhibitor, lowers mitochondrial membrane potential (Δψm), increases ROS generation, and inhibits oxygen consumption and glycolysis while improving insulin sensitivity and promoting WAT browning through the inhibition of KIT, BCR-ABL, and platelet-derived growth factor receptor (PDGFR) [296, 298]. Imatinib, by inhibiting BCR–ABL activity, disrupts the BCR-ABL/PI3K/Akt/FOXO4/ATF5/mTOR pathway and as a result, may induce autophagy [297]. As a proteasome inhibitor, bortezomib reduces the activation of the mTOR pathway, which reduces ER stress, inflammation, and insulin resistance [299]. Although the FDA has approved a number of the aforementioned medications, the practicality of modifying autophagy has yet to be completely proven. Many medications that have metabolic benefits can modulate autophagy; however, autophagy modulation may not be their main/only mechanism of action.

Antidiabetic drugs have modulatory effects on the autophagy process. The cardioprotective effects of sitagliptin were mediated by the stimulation of autophagy [300]. Likewise, sitagliptin promoted autophagy through a method reliant on autophagy regulation, and decreased TG, low-density lipoprotein-cholesterol (LDL-c), and oxidative stress [300, 301]. Sitagliptin activated autophagy by stimulating AMPK and suppressing the mTOR signaling pathway, which in turn had positive effects on hepatic steatosis and insulin resistance [302]. Liraglutide administration by autophagy stimulation inhibited apoptosis and enhanced cognitive function and memory capacity through the suppression of mTOR and the expression of LC-3 II, Beclin-1, PI3K, AMPK, and Akt [300]. By suppressing liraglutide’s protective effects against glucose-induced cardiac injury, the use of 3BDO as an autophagy inhibitor made it clear that mTOR-dependent autophagy was at play [303]. Another antidiabetic drug, exenatide, has hepato-protective effects in diabetic mice, verified by enhanced autophagosome formation and upregulation of Beclin-1 and LC3A/B-II/I [300].

## Conclusion

Given that previous investigations have been conducted on in vitro or animal models, more clinical studies should be performed to better understand the precise role of autophagy and its regulation in insulin resistance, as well as the crosstalk between autophagy and insulin function in varied tissues. Autophagy-targeted therapies provide promising possibilities for the treatment and prevention of insulin resistance. Many medications with good metabolic effects can modify autophagy, but affecting autophagy is not their only mechanism of action.

## Data Availability

Data will be made available on reasonable request.
